# Tracking the Evolution of Smartphone Sensing for Monitoring Human Movement

**DOI:** 10.3390/s150818901

**Published:** 2015-07-31

**Authors:** Michael B. del Rosario, Stephen J. Redmond, Nigel H. Lovell

**Affiliations:** Graduate School of Biomedical Engineering, UNSW Australia, Sydney NSW 2052, Australia; E-Mails: m.delrosario@unsw.edu.au (M.B.R.); s.redmond@unsw.edu.au (S.J.R.)

**Keywords:** smartphone, activity classification, algorithms, sensors, accelerometer, gyroscope, barometer, telehealth

## Abstract

Advances in mobile technology have led to the emergence of the “smartphone”, a new class of device with more advanced connectivity features that have quickly made it a constant presence in our lives. Smartphones are equipped with comparatively advanced computing capabilities, a global positioning system (GPS) receivers, and sensing capabilities (*i.e.*, an inertial measurement unit (IMU) and more recently magnetometer and barometer) which can be found in wearable ambulatory monitors (WAMs). As a result, algorithms initially developed for WAMs that “count” steps (*i.e.*, pedometers); gauge physical activity levels; indirectly estimate energy expenditure and monitor human movement can be utilised on the smartphone. These algorithms may enable clinicians to “close the loop” by prescribing timely interventions to improve or maintain wellbeing in populations who are at risk of falling or suffer from a chronic disease whose progression is linked to a reduction in movement and mobility. The ubiquitous nature of smartphone technology makes it the ideal platform from which human movement can be remotely monitored without the expense of purchasing, and inconvenience of using, a dedicated WAM. In this paper, an overview of the sensors that can be found in the smartphone are presented, followed by a summary of the developments in this field with an emphasis on the evolution of algorithms used to classify human movement. The limitations identified in the literature will be discussed, as well as suggestions about future research directions.

## 1. Introduction

A wearable ambulatory monitor (WAM) or wearable sensor is an electronic device that can be attached to the body or embedded in a clothing garment and is able to record information about the user’s body movements by analysing the signals produced by the device’s transducers. The earliest WAMs consisted of a single uniaxial accelerometer [1] or three orthogonally positioned uniaxial accelerometers [2]. The advent of micro-electro-mechanical systems (MEMS) technology allowed accelerometers and other sensing components to be miniaturised, leading to smaller wearable sensors which contained triaxial accelerometers, triaxial gyroscopes, barometric pressure sensors, and triaxial magnetometers; electronic components that have become commonplace in modern smartphones.

Wearable sensors have garnered much attention in recent decades for their ability to non-invasively estimate energy expenditure [3] and remotely monitor physical movement [4]; all of which are crucial in the evaluation of chronic diseases like diabetes [5] and obesity [6], that can be managed through regular physical movement [7,8]. The analysis of gait has been used to monitor the progression of diseases affecting mobility, such as Parkinson’s disease [9], where WAMs can detect freezing of gait [10]. In the elderly, wearable sensors have found prominence as a means of detecting falls [11] or estimating the likelihood of future falls via gait analysis [12,13].

Bouten *et al.* [2] were pioneers in remote monitoring of physical movement. They conceived a device comprised of a single triaxial accelerometer and data processing unit which could be used to assess physical movement via human body accelerations. Their work established a significant relationship (r = 0.89) between accelerometry and energy expenditure [2], which became the impetus for the wearable sensor revolution that followed. Subsequent work by Najafi *et al.* [14] illustrated that gyroscopes can be used to detect postural transitions and would lead others to use inertial measuring units (IMUs: electronic devices containing a triaxial accelerometer and triaxial gyroscope which can measure both acceleration and angular velocity along three orthogonal axes) to: detect falls using accelerometry signals [15]; incorporate a barometric pressure sensor into a device for fall detection [11]; detect stair ascent and descent with a triaxial accelerometer and barometric pressure sensor [16,17]; non-invasively monitor physical movement with a magnetic and inertial measuring unit (MIMU) [18] and incorporate an IMU into a shoe containing force, pressure, electric field and bend sensors for quantitative gait analysis [19].

Additional information pertaining to the type of physical movement identified could be captured if contextual information about the person’s surroundings was combined with measurements from a MEMS sensor. Ward *et al.* [20] combined a triaxial accelerometer with body-worn microphones to identify nine physical movements in a workshop environment, whilst Rodríguez *et al.* [21] demonstrated that both the speed and positional data from a global positioning system (GPS) receiver could be used to improve algorithms that estimate physical movement in outdoor environments. Concurrent developments in gait analysis that combined knowledge from dead reckoning (a noisy positional estimate due to the error that accumulates when an object’s position is calculated based on both the last known position and estimated velocity during the time interval) with aspects of human biomechanics created a new domain of study, called pedestrian dead reckoning (PDR) [22], that enabled precise estimates of the individual’s position and velocity to be obtained in indoor environments using IMUs. The most reliable estimates could be made when these devices were located on the dorsal surface of the foot, because zero velocity updates (ZUPT) [23] or zero angular rate updates (ZARU) [24] could be performed between each step, allowing for accumulated errors in velocity estimates to be corrected.

These breakthroughs played a pivotal role in establishing WAMs as a means of non-invasively monitoring physical movement; however, their widespread adoption has been limited by the cost associated with purchasing the device, an issue which advances in mobile technology may have inadvertently addressed. The arrival of the smartphone coupled with its ubiquitous nature [25,26] make it the ideal platform upon which to develop a WAM that individuals could elect to use at no additional cost. Smartphone applications designed to replicate the functionality of a dedicated WAM have the potential to assist subsets of the population with the management of a chronic health condition [27] or prevent its onset [28,29]. Individuals who suffer from a health condition that affects gross motor function can use smartphone-based monitors to analyse their gait [30,31] or tremor [32], whilst those who suffer from diabetes or obesity may benefit from an estimate of their energy expenditure [33].

This paper provides a review of the literature related to smartphone-based monitoring of physical movement. A critical analysis of the state-of-the-art in algorithms for smartphone-based monitoring of physical movement are presented, with a focus on the sensors used, features extracted from the sensor signals, movements classified, and the accuracy of each algorithm. Challenges that must be addressed in the future will be identified and possible solutions proposed. In the interests of brevity, the terms accelerometer, gyroscope and magnetometer will refer to tri-axial accelerometers, tri-axial gyroscopes and tri-axial magnetometers, respectively, unless stated otherwise. A smartphone will be defined as the amalgamation of a personal digital assistant (PDA) and mobile phone into a single device [34]. Consequently, those algorithms which were designed for PDAs or mobile devices will be excluded from this review as they have less functionality than smartphones [35]. Whilst the smartphone’s Bluetooth and Wi-Fi connectivity allow it to communicate with countless accessories that increase the smartphone’s sensing capabilities, these sensing modalities require interaction with other devices which are external to the smartphone, and hence will not be discussed further. Instead this review will focus only on sensors whose operation is entirely internal to the device.

The remainder of the paper can be separated into four sections. First, the multimodal sensors that can be found within the smartphone are described, followed by a summary of the state-of-the-art methods by which information about physical movement is extracted from these sensors. A selection of algorithms that illustrate landmark achievements in the state-of-the-art are presented. Comprehensive reviews which focus solely on smartphone-based solutions for fall detection [35] and “online” activity recognition [36] (in which all computation is performed on the smartphone) have been discussed previously. Instead, this paper will review the variety of algorithms that have developed by using the smartphone as a non-invasive physical movement monitor: which sensors can be used to characterise human movement; the breadth of movements that can be identified; as well as how algorithms have evolved to deal with the issue of inconsistent device placement. Finally, the limitations of the state-of-the-art are identified before discussing the challenges that need to be overcome in future work to address these limitations.

## 2. Identifying Physical Movement

**Figure 1:**
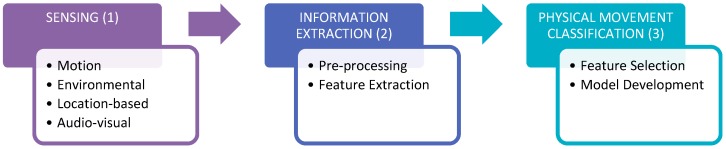
Workflow for identifying physical movement in a smartphone application.

Regardless of the physical movement performed by the user (who is wearing or carrying the device), the movement identification process can be separated into three distinct phases; sensing, information extraction and physical movement identification (as illustrated in Figure 1). During the sensing phase, algorithm designers must decide which sensing components within the smartphone will be utilised. Although it is acceptable to use all of the smartphone’s sensors, the number of sensing components utilised is often minimised to maximize the smartphone’s limited battery life.

### 2.1. Sensing

The smartphone contains multimodal sensors which can be utilised to identify physical movement. Contextual information about the movement can be derived from location sensors (which contain radio frequency components) and audio-visual components, whilst the MEMS sensors experience environmental changes (in the case of the ambient light sensors, barometric pressure sensor and magnetometer) or measure motion due to the movement of the body (in the case of the gyroscope and accelerometer).

#### 2.1.1. Motion MEMS Sensors

Earlier smartphone models contained a single MEMS sensor, an accelerometer. This sensor measures the combined acceleration due to gravity and body movement along three orthogonal axes and enabled existing algorithms [37,38,39] to be implemented as smartphone applications which could estimate the number of steps taken by the user when the smartphone was placed in the pants pocket [40]. Similarly, the incorporation of a six degree of freedom (6DOF) IMU within the smartphone enabled the development of algorithms dependent on the signals from the IMU to be implemented for estimating physical movement. The IMU combines a triaxial accelerometer with a triaxial gyroscope that measures angular velocity along three orthogonal axes and enables changes in the orientation of the device to be determined. In most current smartphones, the IMU would be replaced with a MIMU which incorporated a magnetometer into the IMU.

#### 2.1.2. Environmental MEMS Sensors

**Figure 2:**
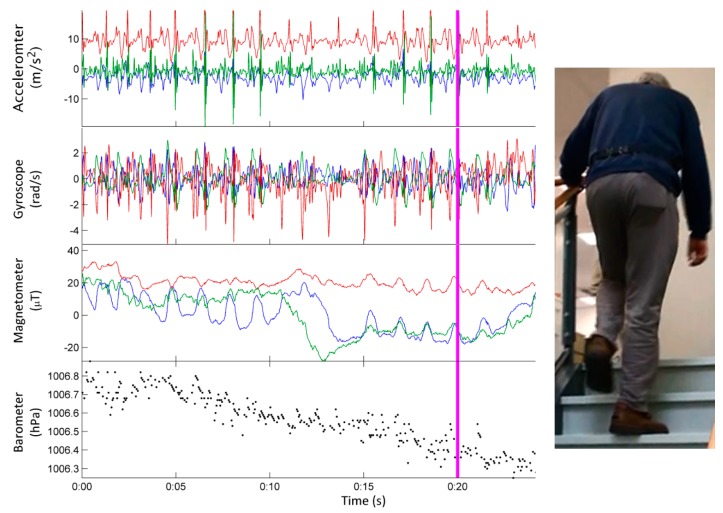
Sensor data stream from the micro-electro-mechanical systems (MEMS) sensors within a smartphone. The accelerometer, gyroscope and magnetometer produce signals along three orthogonally mounted axes: x (blue), y (red) and z (green). The vertical line (magenta) which is present in all of the sensor data streams denotes the time point corresponding to an older adult walking up a staircase whilst a smartphone is placed in the pocket of their pants (image on the right).

The magnetometer measures the strength of the local magnetic field (whose magnitude and direction is influenced by ferromagnetic materials and other magnetic sources in the environment) along three orthogonal axes and can be used to determine the heading of the device relative to the Earth’s magnetic north pole, making it an essential component of a MEMS-based attitude and heading reference system (AHRS) which estimates the device’s orientation (pitch, roll and yaw). Note that the magnetometer sensor has poor frequency response compared to the accelerometer and gyroscope (see Figure 2).

Similarly, the altitude of the device relative to its initial position can be determined from the smartphone’s barometric pressure sensor, which measures absolute atmospheric pressure to infer altitude above sea level (in Figure 2, the measurements from the barometric pressure sensor can be seen to decrease as altitude increases). Whilst the resolution of the barometric pressure sensor is often reported as being accurate to 1 m, the signal may need to be temporally averaged over a number of seconds (e.g., 5 s at 50 Hz) to obtain this level of accuracy [41]. The triaxial accelerometer, triaxial gyroscope, triaxial magnetometer and barometric pressure sensor (see Figure 2) form the central component of non-invasive physical movement monitors due to their ability to sense gross body movement and their relatively low power consumption [42,43] compared to the audio-visual components (*i.e.*, microphone or camera) and location based sensors (*i.e.*, Wi-Fi, Bluetooth) [44].

#### 2.1.3. Location-Based Sensors

Location-based sensors can aid in the identification of physical movement by providing information about the individual’s location. In the smartphone, these sensors include the Wi-Fi transceiver and GPS receiver. Early GPS receivers could perform geolocation (the process of identifying the smartphone’s position on the Earth) in outdoor environments with an accuracy varying between 300 m and 1 km [45], however the current generation of smartphones have built-in GPS receivers that are accurate to within a few metres outdoors [46]. Utilising the device’s GSM module to estimate the distance to cellular base stations within range [47], or the Wi-Fi module to estimate the distance to known access points (within range) can increase the accuracy of GPS-based positional estimates and assists with localisation indoors where GPS signals are ineffective [48]. Although more power-intensive, these two approaches can be combined to improve positional estimates in outdoor environments too.

Liu *et al.* [49] proposed a novel method for improving indoor positioning by incorporating the kinematic motion of the smartphone into the algorithm they developed to estimate the user’s position indoors. Their approach fused information from a PDR algorithm with knowledge obtained by estimating the distance between the smartphone and Wi-Fi access points (whose positions are known) to compensate for the variance in the received signal strength indication (RSSI) at a particular location. Whilst the effects of reflection, diffraction and scattering will impact the propagation of a Wi-Fi signal [50], multipath fading in indoor environments [51] causes the measured RSSI at a particular location to vary about an average value. There is always the risk of violation of personal privacy [52] when tracking a user’s location, an issue which can be exacerbated if the smartphone’s microphone or camera are used, as they may reveal private information about the user and those in close proximity.

#### 2.1.4. Audio-Visual Sensing Components

Lu *et al.* [53] were among the first to identify sound types (such as speech and music) as well as specific sound events (such as driving and climbing stairs) by extracting frequency and time domain features from the smartphone’s microphone. The time domain features they identified were incorporated into an activity classification algorithm by Khan *et al.* [54] that combined information from the smartphone’s microphone, accelerometer and barometer to identify fifteen physical movements. A more power intensive approach involves periodically taking pictures with the smartphone’s camera whilst recording data from the smartphone’s microphone as well as the motion and environmental sensors [55].

Utilising the entire array of the smartphones multimodal sensors enables algorithm designers to harvest more contextual information about the activity when estimating physical movement, however it does reduce battery life, as these contextual sensors are less power efficient than MEMS sensors. Even if the number of sensors used is kept at a minimum, it is still possible to drain the smartphone’s battery in less than one day if the information extraction process is too computationally intensive [56].

### 2.2. Information Extraction

Information extraction encompasses both the data pre-processing and feature extraction methods which are essential to reduce the raw sensor data to a finite number of derived parameters from which a physical movement can be inferred.

#### 2.2.1. Pre-Processing

Pre-processing the MEMS data is of particular importance. Digital filters (*i.e.*, systems that alter discrete-time signals via mathematical operations) are traditionally applied to these signals to improve the signal to noise ratio. Median filters of a short window (*i.e.*, length, n = 3) are often applied to remove spurious noise [4] though they can alter the absolute peak value in the signal [57]. Accelerometer data is typically high-pass filtered to separate acceleration due to gravity (the low frequency component of the signal which can also be used to estimate the orientation of the device) from acceleration due to body movement [58].

Since the smartphone is used for communication and leisure, it will not always be fixed to the body in the same orientation, which can lead to data with high variability with respect to the activity being performed. The issue of inconsistent device orientation has been partially addressed by authors using dimension reduction techniques such as principal component analysis (PCA) [59]; linear discriminant analysis (LDA); kernel discriminant analysis (KDA) [60]; or eigenvalue decomposition [61], to reduce the intraclass variance in the data streams by projecting the normalised accelerations to a global frame in which it is assumed that most of the measured acceleration is due to body movement in the forward-backward plane [61].

Alternatively, the data from an IMU or MIMU can be processed through an AHRS algorithm to estimate the orientation of the device and project the accelerations (measured in the sensor frame) back to the earth frame of reference, which would make the measures robust to inconsistent device orientation. The position of the smartphone in the global frame can then be estimated by double integration of the accelerometry signal, however, the accuracy will be poor without using the zero velocity update technique [23] which relies on the device being worn on the foot. The error associated with the estimated position in the vertical plane can be bound by combining the vertical position estimated from the accelerometer with the relative altitude estimated by a barometric pressure sensor [62]. If the distributions of the noise in the signals from the accelerometer and barometer are known (or approximated) [63], the measurements can be fused to obtain an optimal estimate of the vertical position.

#### 2.2.2. Feature Extraction

Once the data pre-processing is complete, features are usually extracted from sequential epochs of time (each no more than a few seconds long) using a sliding windowing method, often with 50% overlap between consecutive windows [64]. In previous research, features extracted from MEMS signals have shown that the gyroscope and accelerometer signals contain the most information about human movement because they indirectly measure kinematic motion [65]. That being said, the smartphone’s environmental sensors (barometric pressure sensor) can provide supporting information in situations where the physical movement involves rapid changes in altitude (e.g., walking down stairs) or changes in magnetic fields. Furthermore, the smartphone’s microphone allows additional information about the physical movement to be obtained by analysing the audio signal in both the time and frequency domain (see Table 1 for more details) to extract information that may be indicative of motion (such as the rustling of pants or footsteps, or the sound of running water in a kitchen or bathroom) or a particular sporting activity (e.g., the dribbling of a basketball). Time domain features are calculated once for each window, whilst frequency domain features are calculated using all data in the window.

**Table 1:** Features that can be extracted from the smartphone’s sensors to characterise physical movement. The smartphone’s camera is not listed in the table as the images they can produce are often processed with “computer vision” based methods, e.g., speeded-up robust features [66].

Domain	Feature	Accelerometer	Gyroscope	Magnetometer	Barometer	Microphone	GPS/GSM/Wi-Fi
	Position/altitude	✓	✓	✓	✓		✓
**Time**	Signal Magnitude Area [2]	✓	✓				
	Signal vector magnitude [4]	✓	✓	✓			
	Differential pressure [11]				✓		
	Autoregressive coefficients [56]	✓					
	Tilt angle [58]	✓	✓				
	Relative altitude [67]				✓		
	Peak-to-peak amplitude	✓	✓				
	Zero crossing rate [68]	✓	✓			✓	
	Short-time average energy [69]					✓	
**Frequency**	Low energy frame rate [46]					✓	
	Entropy [64]	✓	✓				
	Energy [70]	✓	✓				
	Fast Fourier transform coefficients [71]	✓	✓				
	Discrete cosine transform coefficients [72]	✓	✓				
	Spectral flux [73]					✓	
	Spectral roll-off [74]					✓	
	Spectral centroid [74]					✓	
	Bandwidth [74]					✓	
	Normalised weighted phase deviation [75]					✓	

**Figure 3:**
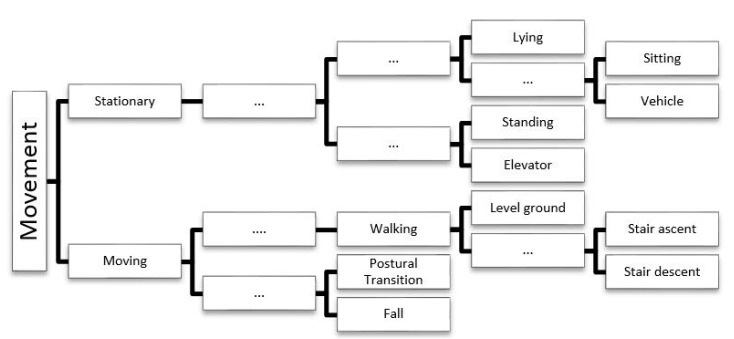
Physical movements in daily living. An example of a hierarchical description of the movements that models built with machine learning algorithms may be able to identify. The “…” symbolises that there may be more than one “intermediate” level before the lowest level of the hierarchy is reached.

Statistical measures [76,77] of the time domain features are calculated for each window so that a finite number of measures containing information about the physical movement during that epoch can be compiled. Time domain features that have previously been calculated for each window include: mean; median; maximum; minimum; standard deviation; variance; skew; kurtosis; root mean square [78]; correlation between axes [70]; interquartile range and percentile [79,80]. The features that are extracted every window are assigned to a category (*i.e.*, the physical movement) that best describes the physical movement that occurred and form the training data that will be used during the development of the classification model [81]. In order to provide accurate descriptions of physical movement, algorithms may utilise a hierarchical description of physical movement (Figure 3) that contain both “higher level” descriptions and “lower level” descriptions of the physical movements that an individual may perform. Training data that is poorly labelled (or has undetailed labels) can lead to models that can only identify movements at a “higher level” (*i.e.*, identify stationary, or moving periods), whilst well-labelled training data (data with detailed labels) can lead to models that can possibly identify movements at a “lower level” (*i.e.*, identify lying, sitting, standing, walking upstairs, walking downstairs, walking on a flat surface, *etc.*). The complexity of the algorithm as well as the level of detail of the training data will determine the degree to which the physical movements can be identified.

### 2.3. Physical Movement Classification

Various models of human movement can be developed depending on the machine learning algorithm (MLA) that is used to analyse the training data. Whilst it may be possible to build a model that it is capable of correctly classifying all of the training data, this can lead to over-fitting which will limit the model’s ability to correctly categorise new/unseen data [82].

#### 2.3.1. Feature Selection

Prior to building a model that will classify human movement, algorithm designers may apply feature selection techniques to the training data to minimise the number of features that are calculated thereby reducing the algorithm’s processing time. In classification problems, such as physical movement identification, feature selection finds the optimal feature subset (from all of the features generated) that can best distinguish between movements. Capela *et al.* [83] recommend using Relief-F, Correlation-based Feature Selection or the Fast Correlation Based Filter because these methods are not biased toward categories that are over-represented in the training data. A comprehensive survey of feature selection algorithms has been compiled by Huan and Lei [84]; all of which can be used in the evaluation of features extracted for the identification of physical movement.

#### 2.3.2. Model Development

At this point, the model which will ultimately identify the physical movements can be developed. Initially, these models were limited to heuristic classifiers due to the smartphone’s limited processing capabilities; however, the modern smartphone has sufficient computational capability to execute state-of-the-art feature extraction algorithms and classification models. These models are trained using an MLA which will analyse the training data during a supervised learning process to define a function (or series of rules) which can be used to separate the training data according to the different assigned categories (in this case physical movement types) whilst minimising the number of misclassifications.

In many cases the Waikato Environment for Knowledge Analysis (WEKA) [85,86] data mining toolbox, has enabled these MLAs to be seamlessly implemented on the smartphone because they were written in the Java programming language which is the primary language used when developing smartphone applications for the Android platform. Preece *et al.* [87] provides a thorough summary of the MLAs (listed in Table 2) that can be used to build models for identifying physical movement. Accepted methods for validating the models include (but are not limited to): leave-one-out cross-validation; the k-fold cross validation method; or the ‘.632+’ bootstrap method [88]. The type of physical movement identifiable using the smartphone’s internal sensors is dependent on how the smartphone is worn on the body and the granularity of the collected training data (*i.e.*, the variety of activities and the number of instances of each activity). In the absence of a belt or clip to fix the smartphone to the body, it is difficult to distinguish between sport-specific movements and those that a person might perform as they move throughout the day because the orientation of the device relative to the body cannot be assumed to be constant. This would make it difficult to distinguish the movement of a seated individual swinging their arms whilst holding a smartphone in-hand from walking whilst the smartphone was placed in the pocket of their pants.

**Table 2:** Examples of machine learning algorithms which can be used to build models that can classify human movement.

Machine Learning Algorithms (MLAs)		
Hidden Markov Models (HMM)	K Nearest Neighbours (KNN)	Support Vector Machines (SVM)
Bayesian Networks (BN)	Gaussian Mixture Models (GMM)	Logistic Regression (LR)
Naïve Bayes (NB)	Decision Tree Classifiers (DTC)	Artificial Neural Networks (ANN)

If the smartphone is fixed to the body (e.g., belt, armband, wrist strap) or is placed in a custom apparatus (e.g., balance board) the movements can be specific to a particular sport [89,90] or exercise [91,92]. In certain circumstances, the movement measured by the sensors at some body positions may not contain enough information to distinguish between different physical movements (e.g., sensors placed on the trunk will struggle to differentiate between standing and sitting.) which can limit the number of identifiable movements that a model (which is built using this training data) will be able to identify.

## 3. Limitations Leading to Algorithm Evolution

A major limitation of using smartphones to estimate human movement is that the device’s multifunctional nature ensures that it will not be consistently located and/or oriented at the same position on the body. Consequently, algorithms for monitoring physical movement have evolved to address the limitations of prior art. The initial generation of algorithms required the smartphone to be firmly fixed to the body at a known location with a strap, armband or custom apparatus (Figure 4a). This constraint was relaxed in the second generation of algorithms which allowed the smartphone to be placed in a specific pocket of the user’s clothing (Figure 4b), a constraint which was removed in the current state-of-the-art (Figure 4c).

**Figure 4:** Evolution of smartphone algorithms with respect to where they are carried: (**a**) assume the smartphone is firmly fastened to the body at a known location; (**b**) assume that the smartphone is located in the same area on the person’s body (whether it is held in the hand, placed in the chest or trouser pocket) throughout the day, but its orientation need not be fixed with respect to the body; (**c**) “state-of-the-art” algorithms are capable of identifying physical movement whether the device is held in the hand, or in a pocket of the individual’s clothing.

**Table:** 

Fixed to the body (a)		Body position dependent (b)		Body position independent (c)
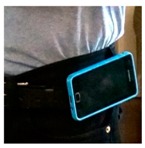	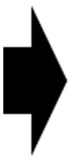	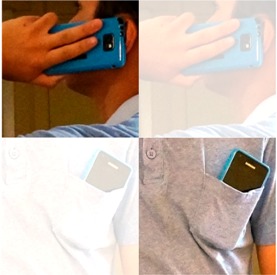	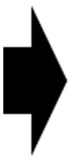	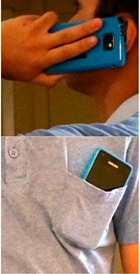

### 3.1. Fixed to the Body Algorithms (FBAs)

Early algorithms required the smartphone to be fixed to the body with a strap or additional apparatus at a predetermined position, which ensured that the relative orientation of the smartphone to the body is fixed [93] necessitating only the use of “digital filters” during the pre-processing stage. This enabled features to be extracted that are derived from biomechanical principles unique to the point of fixation (e.g., tilt angle) which can then be exploited by the MLAs in the development of a model that can identify specific body movements. If the goal is to characterise gross body movement, the device should be positioned close to the body’s centre of mass [94]. The lower back is closest [95] to the body’s centre of mass (just above/below the level of the navel), which has led researchers to place the smartphone on a belt at this position. Shumei *et al.* [96] used the ensemble learning (*i.e.*, they combined two MLAs, in this case a DTC and SVM) approach and the smartphone’s accelerometer to identify six physical movements that only required the device to be fixed on the left side of the waist with a belt. Improvements to their approach were made by using a smartphone which contained an IMU [97] and eventually a MIMU [77], thereby enabling a wider variety of movements to be identified (for more details see Table 3) and allowed estimates of energy expenditure based on metabolic equivalents [98] to be calculated. Similarly, smartphone applications which detect simulated falls are most reliable when the device is located near the body’s centre of mass because this position is in a near free-fall state (*i.e.*, the measured acceleration will be close to 0 m·s^−2^) [99].

Physical movements unique to a particular sport or activity can be identified if the smartphone is placed in a custom apparatus. Mitchell *et al.* [90] created a vest with a pouch between the scapulae where the smartphone could be placed and models developed utilising MLAs (*i.e.*, SVM, KNN, ANN, NB, DTC) to identify seven movements specific to the sports of field hockey and soccer. In this scenario, the smartphone would enable the team’s physical trainers to quantify their athletes’ performance.

Waterproof cases have expanded the reach of smartphone technology to marine environments. Marshall [89] devised a prototype application capable of tracking the absolute position, posture and velocity of swimmers (in the pool) by attaching the waterproof case at the lower back, with a strap around the waist. It is hoped that this application could ultimately serve as a surrogate “swimming coach”, capable of providing users with feedback after their practice session.

The use of smartphone technology as a coach or personal trainer has also been explored in the more traditional environment of the gym. Kranz *et al.* [91] developed a personal training application that could provide users with feedback on twenty exercises performed whilst a smartphone was mounted to the center of a balancing board. The application analysed data obtained from the smartphone’s motion sensing MEMS (*i.e.*, the accelerometer) and environmental sensing MEMS (*i.e.*, the magnetometer) to provide feedback on how the user performed each exercise.

Pernek *et al.* [100] devised a similar model that used logistic regression to monitor weight training programs in a gym. Their application counted the number of times different muscle groups were exercised but required users to place the smartphone in a strap for the ankle or wrist and specify which muscle group (biceps brachii, gluteus maximus, hamstrings, latissimus dorsi, pectoralis major, quadriceps, rectus abdominis, soleus, and triceps brachii) was being exercised. Although users would have to continually move the smartphone between ankle and wrist as they exercised, it could determine the number of repetitions performed regardless of whether the individual was using a machine in a gym or free weights and resistance bands. This limitation was overcome by Muehlbauer *et al.* [92], whose model for identifying physical movement could distinguish ten upper body exercises, count the number of times each exercise occurred and distinguish these physical movements from other movements whilst in the gym (drinking water, walking, *etc.*).

All of these algorithms require the smartphone to be firmly fixed to the body, often with a strap that is not commercially available [91,92,100,101] and can be restrictive to wear [77,90]. Additionally, some manufacturers have increased the device’s screen size from an initial size of 3.5” (1st generation iPhone) or 3.7” (Nexus One) to 4.7” (iPhone 6) or 5.1” (Samsung S6) which would make them more cumbersome to wear, but is catering to user demand for the device as medium for video. In either case, these apparatuses may not be representative of how people carry their smartphone throughout the day.

**Table 3:** Applications where the smartphone has been used as a substitute for a dedicated wearable ambulatory monitor (WAM).

Author	Application	Sensors	Placement	Subjects (M:F)	Machine Learning Algorithm	Movements	Outcome
**Joundi *et al.* [32]**	Rapid tremor assessment	Accelerometer	Strapped to the forearm or lower leg	7 (undisclosed)	Undisclosed	Tremor (multiple sclerosis, essential tremor, post-stroke, dystonic, Parkinson’s Disease )	iPhone accelerometer can be used to identify the dominant tremor frequency
**He and Li [77]**	Physical movement, simulated falls	MIMU	Placed in a strap over the chest	10 (6:4)	Hierarchical classifier comprised of 14 binary classifiers.	Sitting, lying, standing, postural transitions, walking, stair ascent and descent, running, jumping, falling (forward, backward, to the left or right)	Accuracy 95.03%
**Marshall [89]**	Swim coach	MIMU	Placed on the lower back	Undisclosed	Undisclosed	Body posture, swim velocity	Undisclosed
**Mitchell *et al.* [90]**	Athletic performance during five-a-side soccer and field hockey	Accelerometer	Inserted into vest, between scapulae	32 (undisclosed)	Wavelet transform, NB, KNN, ANN, DTC, SVM	Stationary, walking, jogging, sprinting, hitting the ball, standing tackle, dribbling	F_soccer_ = 0.799 F_hockey_ = 0.823
**Kranz *et al.* [91]**	Physical exercise trainer	Accelerometer, magnetometer, Wi-Fi, 3G	Placed in the centre of a balance board	6 (undisclosed)	Pyramidal principal component breakdown analysis	Static and dynamic balancing exercises performed on a balancing board (20 in total)	r_dynamic_ = 0.549 r_static_ = 0.771
**Muehlbauer *et al.* [92]**	Detect specific upper arm exercises and the number of repeats	IMU	Holster on upper arm	7 (6:1)	KNN	Butterflies, chest press, latissimus, abdominal, upper back, shoulder press, pulldown, low row, arm curl or extension	Accuracy 85.1%
**Tacconi *et al.* [95]**	Simulated falls	Accelerometer	Belt-worn on the waist over the lower back	3 (undisclosed)	Single-threshold based algorithm	Various falls: forward, lateral, backward, sliding against a wall, out of bed	Detected 65 out of 67 simulated falls
**Shumei *et al.* [96]**	Physical movement	Accelerometer	Belt-worn on the left side of the waist, landscape orientation.	10 (7:3)	DTC SVM	Sitting, standing, lying, walking, postural transitions, gentle motion	Accuracy 82.8%
**Anguita *et al.* [97]**	Physical movement	IMU	Placed in a belt on the waist	30(undisclosed)	SVM	Standing, walking, sitting, lying, stair ascent, stair descent	Accuracy 89%
**Aguiar *et al.* [98]**	Physical movement, estimate energy expenditure	Accelerometer	Placed in a belt, worn on the waist	31 (21:10)	DTC	Lying, standing, walking, random, running	Accuracy 99.4%
**Pernek *et al.* [100]**	Exercise repetition detection using exercise machines in a gym or free weights and resistance bands.	Accelerometer	Placed on the exercise machines weights; attached to the wrist or ankle.	10 (6:4)	Logistic regression	Squats, leg curl, leg extension, calf raise, triceps extensions, bicep curls, abdominal crunches, bench press	F = 0.993 ± 0.034
**Stöggl *et al.* [101]**	Cross-country skiing	Accelerometer	Smartphone strapped to chest (portrait orientation)	11 (7:4)	Markov chain of multivariate Gaussian distributions	Skating techniques G2, G3. G4. G5	Accuracy 100%
**Mellone *et al.* [102]**	Simulated falls	MIMU	Placed in a belt and worn on the waist over the lower back	Undisclosed	Threshold of 2.3 g on the acceleration sum vector	Timed-up-and-go test, fall detection	Validation of smartphone’s ability to host another process for fall monitoring.

### 3.2. Body Position Dependent Algorithms (BPDAs)

Body position dependent algorithms (see Table 4) require the smartphone to be placed in a consistent location (e.g., pants or shirt pocket) but do not need the device to be firmly fixed to the body in a specific orientation, an issue which can be overcome by utilising pre-processing techniques, in particular dimension reduction (PCA/LDA/KDA). The primary advantage of these algorithms over FBAs is that they are not reliant on the user purchasing additional equipment to estimate physical body movement. This has led to the emergence of algorithms capable of estimating physical movement whilst the phone is held in the hand, or is placed in the pants or shirt pocket.

**Table 4:** Applications where a smartphone has been used as a body position dependent WAM.

Author	Application	Sensors	Placement	Subject total (M:F)	Machine Learning Algorithm	Movements	Outcome
**Guidoux *et al.* [33]**	Physical movement, estimate energy expenditure	Accelerometer	Pants pocket	12 (6:6) directed 30 (15:15) free-living	Periodic function peak	Sitting, normal and brisk walking, stair ascent, stair descent, standing, slow running, riding a tram	Accuracy of 73.3% ± 10.3%
**Shoaib *et al.* [65]**	Physical movement	MIMU	Pants pocket, chest pocket, lateral surface of the bicep, wrist	10 (10:0)	DT, KNN, BN, NB. SVM, LR	Stair ascent, stair descent, walking, jogging, biking, sitting, standing	Gyroscope can detect stair ascent/descent with greater accuracy than accelerometer. Jogging, walking and running identified at comparable rates with either gyroscope or accelerometer
**Aguiar *et al.* [98]**	Physical movement, estimate energy expenditure	Accelerometer	Pants pocket	31 (21:10)	Decision tree algorithm	Sitting, standing, walking, running, random	Accuracy of 99.5%
**Albert *et al.* [103]**	Physical movement	Accelerometer	Pants pocket	18 (6:12) 8 (1:7)	SVM, sparse multinomial LR	Walking, standing, sitting, holding the phone whilst standing (arms bent forward), holding phone placed on table	The model trained on data from 18 healthy individuals could only predict the activity of the 8 individuals with Parkinson’s disease with an accuracy of 60.3%
**Pei *et al.* [104]**	Physical movement	MIMU	Pants pocket	4 (undisclosed)	Least squares SVM	Sitting, walking, fast walking, standing, sharp turning (>90°), small turns (	Accuracy 92.9%
**Del Rosario *et al.* [105]**	Physical movement	IMU, barometer	Pants pocket	57 (40:17)	J48 DT	Standing, sitting, lying, walking, stair ascent, stair descent, postural transitions, elevator up, elevator down	Data from older cohort can be used to build a decision tree based classifier that is more robust at estimating activities of daily living from different age groups.
**Varnfield *et al.* [106]**	Count steps, physical activity level	Accelerometer	Belt on waist	11 (7:4)	Undisclosed	Count steps, activity level: (sparse, moderate, high, intense)	Step counter <2% error rate in controlled environment

Bylemans *et al.* [107] used the accelerometer within a smartphone to detect steps, and dynamically estimate step length based on the height and gender of the user. The accuracy of their model was affected by the magnitude of the impact accelerations measured due to the heel striking the floor during ambulation, causing those who tread more lightly or heavily to have their step length underestimated or overestimated (respectively) because the model assumed that larger accelerations during ambulation are indicative of bigger steps. Similarly, Pratama *et al.* [108] evaluated the accuracy of three step length estimators (Weinberg [37], Kim *et al.* [38] and Scarlett [39]) which assumed that the phone was held in the hand with the screen facing the user during walking on level ground. The method proposed by Scarlett [39] produced the lowest error in total distance travelled.

Ayub *et al.* [109] benchmarked the same step length estimators (Weinberg [37], Kim *et al.* [38] and Scarlett [39]) under three different “carrying modes” (in pants pocket, held in hand, or held next to the ear) and again found that the step length estimation method proposed by Scarlett [39] produced the smallest error. Combining these estimates of walking distance with models (developed from MLAs) that characterise physical movement [65,103,104,105] will facilitate long-term, non-invasive monitoring, enabling clinicians and healthcare professionals to determine if patients are maintaining a healthy lifestyle [110] whilst analysing gait with dedicated wearable ambulatory monitors has been shown to offer insight into the physical health of patients [111,112,113].

Antos *et al.* [93] identified the most important limitation of BPDAs when they demonstrated that the accuracy of models developed with a MLA (*i.e.*, SVM and HMM) drop significantly if they are used to estimate physical movement while worn at an alternative location on the body. In the analysis they performed, the accuracy of the model used to identify five physical movements (standing, sitting, walking, transition between sitting and standing) from data collected with a smartphone at four different body locations (hand, belt, pants pocket or bag) dropped; from 90.8% when the location of the smartphone was known; to 56.8% when data collected from all positions were classified by a model that was trained on data obtained from only one position. Whilst models dependent on device position should not be used in critical contexts, for example, where a person’s health or welfare may be dependent on the model’s accuracy (e.g., fall detection algorithms [114]), they are adequate for estimating physical movement provided that the user places the device at the same location on their body.

### 3.3. Body Position-Independent Algorithms (BPIAs)

Throughout the day the user may move the smartphone from one location on the body to another, depending on what they are doing. To account for this variability, models (see Table 5) needed to be developed that considered smartphone position on the body [93] before determining the physical movement. Incorporating this logic into existing smartphone applications increases their ability to identify the correct movement regardless of the device’s position on the body, but results in increased computational complexity because the information extraction phase may require the use of digital filters and dimension reduction techniques before estimating where the device is positioned on the body and attempting to identify the physical movement. Additionally, it may reduce the granularity of the identifiable physical movements (*i.e.*, the model may not be able to identify all of the movements listed at the lowest level of the hierarchy in Figure 3).

Anjum and Ilyas [115] proposed a model capable of identifying seven physical movements, although it was not able to identify the three stationary postures (standing, lying or sitting) or transitions between them (recall Figure 3). This would inhibit the application’s ability to estimate the individual’s risk of cardiovascular disease, which has been shown to be linked to prolonged periods of sitting [116,117,118,119]. Antos *et al.* [93] was able to distinguish standing and sitting but could not identify ambulatory movements associated with walking on stairs, which expends more energy compared to walking on a level surface [120]. Henpraserttae *et al.* [61] could identify the three primary postures but also failed to identify stair ascent and descent.

**Table 5:** Applications where a smartphone has been used as a position independent WAM.

Author	Application	Sensors	Placement	Subject Total (M:F)	Machine Learning Algorithm	Movements	Outcome
**Khan *et al.* [54]**	Physical movement	Accelerometer, barometric pressure, microphone	Front and back pants pocket, jacket breast pocket	30 (18:12)	Kernel discriminant analysis, SVM	Walking, walking on treadmill, running, running on treadmill, stair ascent, stair descent, elevator up, elevator down, hopping, riding a bike, inactive (sitting or standing), watching TV, vacuuming, driving a car, riding a bus	Accuracy 94%
**Khan *et al.* [56]**	Physical movement	Accelerometer	Front and back pants pocket, jacket breast pocket	10 (6:4)	ANN	Standing, walking, running, stair ascent, stair descent, hopping	Accuracy 86.98%
**Henpraserttae *et al.* [61]**	Physical movement	Accelerometer	16 orientations on the waist, shirt and pants pocket	10 (undisclosed)	KNN (k = 3)	Lying, sitting, standing, walking, running, jumping	Accuracy 86.36%
**Antos *et al.* [93]**	Physical movement	IMU	Hand, belt on waist, pants pocket, backpack	12 (undisclosed)	SVM, HMM	Standing, sitting, walking, transitions between sitting and standing	Accuracy 87.1%
**Anjum and Ilyas [115]**	Physical movement	IMU	Hand, pants pocket, handbag, shirt pocket	10 (undisclosed)	J48 DT	Walking, running, stair ascent, stair descent, driving, cycling, inactive	Accuracy 94.39%
**Lu *et al.* [121]**	Physical movement	Accelerometer, microphone, GPS	Pants pocket (front/back), hand, armband, backpack, belt, jacket breast pocket	16 (12:4)	SVM	Stationary, walking, cycling, running, vehicle	Accuracy 95.1%
**Han *et al.* [122]**	Physical movement, context recognition	Wi-Fi, GPS, microphone, accelerometer	Waist, pants pocket or hand	10 (undisclosed)	HMM, GMM	Walking, jogging, inactive, bus moving, bus (traffic jam) bus stationary, subway moving, subway stationary	Accuracy 92.43%
**Sun *et al.* [123]**	Physical movement	Accelerometer	Pants pocket (front/back), 4 different orientations in the front pants pocket, blazer front pocket	7 (6:1)	SVM	Stationary, walking, running, cycling, stair ascent, stair descent, driving	F-score 93.1%
**Thiemjarus *et al.* [124]**	Physical movement	Accelerometer	Placed on the waist, in the shirt and trouser pocket	8 (6:2)	KNN (k = 1)	Lying, sitting, standing, walking, running, jumping	Accuracy 75.19%

The advantage of position-independent algorithms over those previously described is that they allow the user to use their smartphone as they would otherwise, without restriction. A recent study by Wang *et al.* [125] remotely monitored forty-eight university students over a period of ten weeks, requiring participants to simply install an application, *StudentLife*, on their smartphone. The application they developed combined their previous work [53,121,126] to detect physical movement regardless of device position and orientation. Whilst their application could only detect periods of physical activity in windows of ten minutes, they were able to use the measurements they obtained from the smartphone to show a significant negative correlation between total activity time per day and loneliness (p=0.018). This finding, along with recent work by Varnfield *et al.* [106] in which the smartphone’s sensing capabilities are used to remotely count the steps of patients undergoing cardiac rehabilitation within their own homes, illustrate the vast potential that smartphones have to improve healthcare if they are utilised as a non-invasive monitor of physical movement.

## 4. Challenges

**Figure 5:**
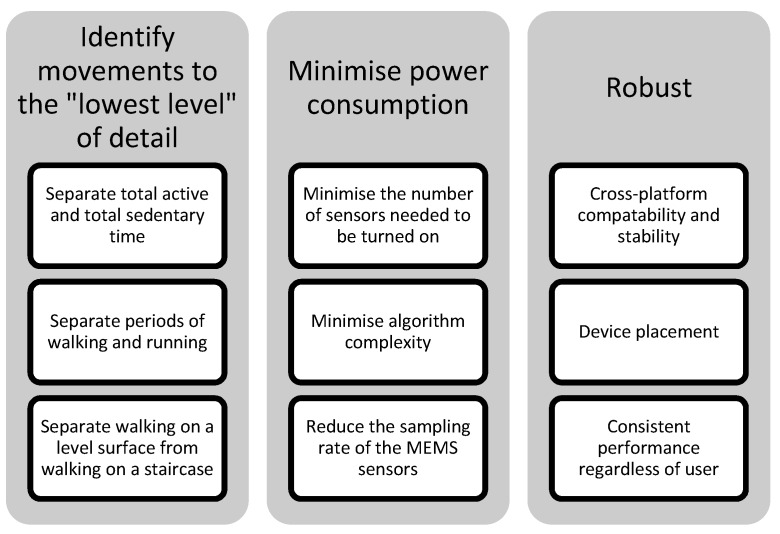
Properties of the ideal algorithm for physical movement identification. Examples of what an ideal algorithm would need to achieve to satisfy each criterion are listed below each property.

There are a number of design criteria which need to be addressed when algorithms for estimating physical movement are developed (see Figure 5). Ideally the algorithm would consume a minimal amount of power whilst accurately classifying physical movements to the “lowest level” of detail (see Figure 3), regardless of the smartphone, its position on the body, its orientation and the physical characteristics of the user. The challenges that prevent these criteria being satisfied by a single algorithm will be discussed before outlining future avenues that warrant further investigation.

### 4.1. Smartphone Battery Life

**Figure 6:**
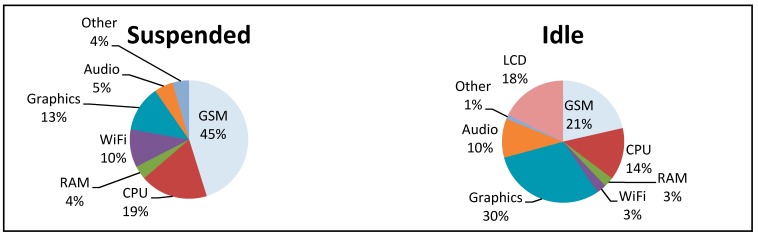
Power consumption of various smartphone hardware components. Adapted from Carroll and Heiser [43].

The limited battery life of the smartphone is a prominent issue which users deal with by regularly charging their device, or charging when they receive a notification from the device to do so [127]. Falaki *et al.* [42] identified that the rate at which the smartphone’s battery is consumed is dependent on three factors: (i) the number of user interactions with the device; (ii) the applications installed and run by the user on the device; and (iii) the hardware and operating system within the device. Carroll and Heiser [43] determined that in a “suspended” state (no applications running/screen switched off) much of the Android smartphone’s battery power (OpenMoko Neo Freerunner) is consumed by the GSM module, whilst in an “idle” state (screen on but no applications running) the graphics processor consumes the most power (a more detailed breakdown can be found in Figure 6). In an attempt to determine which hardware components consume the most power, they performed a number of use case scenarios, to assess which hardware components consumed the most power under varying circumstances. Among their findings, Carroll and Heiser identified that even modest smartphone usage (using the device to call; SMS; browse the internet; and email for a total of three hours) could reduce the battery life to twenty-one hours.

The bigger displays which have become prominent in modern smartphones require more power and have exacerbated battery life problems [128,129]. This limitation led Khan *et al.* [56] and Han *et al.* [130] to develop computationally “lightweight” algorithms (*i.e.* feature extraction methods and models that do not require large amounts of memory or relatively complex mathematical operations) that could estimate physical movement whilst minimising the rate at which the smartphone’s battery was consumed.

The rate at which the smartphone’s battery is depleted will be affected by internal and external sensing characteristics. Internal sensing characteristics are sensor-dependent and include the sampling rate and resolution mode of the sensor (in the case of the motion and environmental MEMS sensors). The resolution mode of the sensor can dramatically alter the rate of power consumption, particularly if the barometric pressure sensor is used. The LPS331AP from STMicroelectronics (found in the Samsung Galaxy S3) consumes 5.5 μA in its low resolution mode, but consumes 30 μA in its high resolution mode. Similarly, the BMP180 from Bosch Sensortec (found in the Samsung Galaxy Nexus and Nexus 4) consumes 3 μA in the ultra-low power mode and 32 μA in the advanced resolution mode if the sensor is sampled at 1 Hz.

Conversely, external sensing characteristics are related to the number of active sensors and the computational complexity of the information extracted from the data (*i.e.*, during the pre-processing and feature extraction stages). Abdesslem *et al.* [44] showed that a 1200 mAh battery could power a smartphone (Nokia N95) for 170 h with all sensors switched off, whilst Khan *et al.* [56] demonstrated that physical activity could be estimated with sampling rates as low as 20 Hz without compromising classification accuracy and recommended the use of time-domain features over frequency domain features as their calculation consumed less power. Additionally, further reductions in energy consumption could be attained by reducing the number of features extracted as well as the computational complexity of the features [56,131]. Algorithm energy efficiency will continue to be an important criterion that needs to be considered, unless advancements in battery technology lead to higher density energy storage, or methods for reducing the power consumption of hardware components are developed [44]. Such methods could include power management algorithms that dynamically change which sensing components are switched on, or change the sampling rate of powered-on MEMS sensors based on battery life or magnitude of acceleration/angular velocity.

### 4.2. Algorithm Granularity

In order to develop algorithms that can identify physical movements to the “lowest level” of detail (recall Figure 3), the notion of energy efficiency is largely disregarded, since data from all of the available sensing components must be analysed in combination [49,104,122,130]. The granularity to which physical movement can be estimated is inherently dependent on where the device is located on the body and how firmly it is fixed at that location. If the smartphone is placed in the pants pocket, it can separate the physical movements of walking and running [132], but may struggle to distinguish bicep curls from tricep extensions. This will have implications on energy expenditure estimates which will be unable to account for increased energy expenditure due to carrying a load (realistically it is not possible for any wearable sensors worn on the lower body to estimate energy expenditure due to upper body movement); or performing an activity which consists primarily of upper body movement [133] (when the smartphone is placed on the waist or in the pants pocket); or deviations in energy expenditure due to changes in walking surface (such as moving between sand and a firm-surface) [134]. Additionally, the accuracy of the estimated energy expenditure has been shown to deteriorate with increased work rate due to a combination of moving at greater velocity or on an incline [3].

### 4.3. Algorithm Robustness

#### 4.3.1. Cross Platform Variability

Ideally, a single algorithm would be developed that could be deployed across all smartphone handsets. This is not a trivial exercise due to differences in the smartphone operating system (OS), software and hardware which vary across platforms. Yanyan *et al.* [135] identified the three options (*native*, *web*, *hybrid*) that software developers can choose between when developing an application for the smartphone and noted that whilst native applications lead to better application performance, it is more difficult to migrate these applications from one platform to another compared to web or hybrid applications. Additionally Android, MeeGo, and Symbian platforms allow any application to run in the background (*i.e.*, applications that continue to run even when they are not visible on the smartphone’s screen), whilst iOS does not support applications that continuously access the inertial sensors in the background [136]. This difference can limit the type of applications that are suitable for cross platform development.

Whilst the MEMS sensing components of many smartphone devices are comparable, the operation mode (sensor resolution and range of operation) vary considerably across devices and is a critical issue which would prevent the widespread adoption of a single algorithm. Table 6 lists the specifications of the MEMS sensors (both motion and environmental) in some currently available smartphone devices, and clearly illustrates that the range of operation and resolution are not consistent across devices. This limits the portability of algorithms developed with data obtained from one smartphone and deployed on another device with a different sensor set or operation modes, since measurements across devices will not be comparable, particularly if the sensors reach their saturation value. Habib *et al.* [35] reported that the range of operation of smartphone accelerometers (±2 g; g = 9.81 m·s^−2^) may ultimately prove too narrow for the purposes of fall detection; however this limitation is due to an inability to change the sensor’s resolution. Whilst it is not yet possible to change the sensor’s range of operation, the newest commercially available smartphones (HTC One, iPhone 6/6+) have accelerometers with a range of operation far exceeding ±2 g, making these devices more suitable for detecting falls, where the magnitude of the impact acceleration can reach values of 3.5 g [137], but at the cost of reduced resolution.

**Table 6:** Sensor specifications of some commercially available smartphones. Note the following abbreviations: accelerometer (A), gyroscope (G), magnetometer (M), barometer (B), g = 9.81 m·s^−2^.

	A	G	M	B	Range	Resolution
Galaxy Nexus	✓				±2 g	±0.61 m·s^−2^
		✓			±2000 °/s	±0.06 °/s
			✓		±800 μT	±0.15 μT (x/y axis) ±0.30 μT (z axis)
				✓	300–1100 hPa	±1 hPa
HTC One	✓				±4 g	±0.039 m·s^−2^
		✓			±2000 °/s	±0.06 °/s
			✓		±4900 μT	±0.15 μT
Samsung S4	✓				±2 g	±0.001 m·s^−2^
		✓			±500 °/s	±0.057 °/s
			✓		±1200 μT	±0.15 μT (x/y axis) ±0.25 μT (z axis)
				✓	300–1100 hPa	±1 hPa
Samsung S3	✓				±2 g	±0.01 m·s^−2^
		✓			±500 °/s	±0.015 °/s
			✓		±1200 μT	±0.30 μT
				✓	260–1260 hPa	±0.24 hPa
Samsung S2	✓				±2 g	±0.002 m·s^−2^
		✓			±2000 °/s	±0.06 °/s
			✓		±1200 μT	±0.30 μT
iPhone 6/6+	✓				±8 g	±0.002 m·s^−2^
		✓			±2000 °/s	±0.06 °/s
			✓		±4900 μT	±0.15 μT
				✓	300–1100 hPa	±0.16 hPa
iPhone 5/5s	✓				±8 g	±0.002 m·s^−2^
		✓			±2000 °/s	±0.06 °/s
			✓		±1200 μT	±0.30 μT
iPhone 4/4s	✓				±2 g	±0.002 m·s^−2^
		✓			±2000 °/s	±0.06 °/s
			✓		±1200 μT	±0.30 μT
LG Nexus 4	✓				±4 g	±0.001 m·s^−2^
		✓			±500 °/s	±0.015 °/s
			✓		±4912 μT	±0.15 μT
				✓	0–1100 hPa	±1 hPa

#### 4.3.2. Device Placement

Smartphones will continue to be an attractive platform for identifying physical movement so long as they are placed on the body. Whilst dimension reduction techniques (see Section 2.2.1) have been employed to normalise data of high variability due to inconsistent device orientation, the data from a MIMU has yet to be fused via an AHRS algorithm for the purposes of identifying physical movement. Utilising an AHRS would enable local frame accelerations to be projected back to the global frame of reference, enabling features to be derived that are independent of device orientation and may lead to new features based in biomechanics, akin to the principals which govern the ZARU and ZUPT used in pedestrian dead reckoning.

Habib *et al.* [35] noted that individuals may not place their smartphone in a garment of clothing that they are wearing whilst at home, limiting the ability of the smartphone to monitor physical movement in the home. That being said, older adults have shown a preference to place a smartphone in a pocket of their clothing rather than wear a dedicated device [138] when given the option, a preference which may extend to other subpopulations when asked to wear a non-invasive monitor for their health. At present, it is unclear if methods need to be developed to identify these periods when the smartphone is not placed on the body, as well as developing mechanisms or strategies to facilitate non-invasive monitoring during these times.

#### 4.3.3. Inter-Subpopulation Predictive Capability

Albert *et al.* [103] identified a model’s inability to correctly classify movements across subpopulations that performed the same physical movements when they trained an algorithm for quantifying physical movement on eighteen healthy individuals (33.0 ± 4.5 years) and evaluated its predictive capability in a cohort of eight individuals with Parkinson’s disease (67.0 ± 8.1 years). They discovered that the algorithm could not predict the movements to the same degree of accuracy within the group suffering from Parkinson’s disease. This inability to predict physical movements across cohorts with different physical/physiological characteristics was independently identified by Del Rosario *et al.* [105] who found that an algorithm trained on older adults (83.9 ± 3.4 years) was better able to predict physical movements in younger adults (80.5% ± 6.8%) than an algorithm trained on younger adults (21.9 ± 1.7 years) and tested on older adults (69.2% ± 24.8%).

These findings have consequences if the classifier forms the basis of an algorithm designed to estimate energy expenditure due to the link between metabolic equivalents (METS) and physical activity [139]. Guidoux *et al.* [33] incorporated this approach into an algorithm for estimating the total energy expenditure (accurate to within 10%) which they tested in a group of young adults (34.1 ± 10.5 years). They acknowledged that these estimates would not be generalisable to a population of young children or obese adults due to differences in behaviour during physical movement.

### 4.4. Future Research

#### 4.4.1. New Sensing Components

Smartphones will continue to change as emerging technologies are miniaturised before being integrated into the “next-generation” device. Comparing the first generation of smartphones with those that are representative of the current “state-of-the-art” it is evident that future devices will contain more sensing components (a fact that is self-evident), all of which may aid in the identification of physical movements. Whilst algorithms have been developed which use the smartphone’s camera and flash to estimate heart rate/pulse [140,141], the advent of a dedicated “heart-rate” sensor within the latest iteration of smartphones (e.g., Samsung S5) provides further evidence that more sensors which were initially restricted to the healthcare sector may eventually be found within smartphone devices. In this domain, future research efforts should focus on identifying sensors that might be suitable for integration into the next generation of smartphones, as well as developing algorithms that incorporate these sensing components to improve existing algorithms for identifying human movement.

#### 4.4.2. MEMS Sensor Management

“Smart” algorithms that are able to dynamically alter the sampling rate of the smartphone’s MEMS sensors need to be investigated and developed to maturity. These smart algorithms would decrease the sampling rate of the MEMS sensors when the individual is determined to be sedentary, disable the MEMS sensors completely when the smartphone is not located on the person’s body and increase the sampling rate when the person is active. Methods should also be developed to change the sensor’s range of operation as this may aid in the identification of certain physical movements which are difficult to discriminate, particularly when the accelerometer is in the ±2 g range of operation mode [137].

#### 4.4.3. Convergence with Emerging Technologies

The arrival of the “smartwatch” as well as the inevitable development of other “smart” garments of clothing which are able to communicate with the smartphone will present an opportunity for algorithm designers to identify (with greater certainty) both where and when the smartphone is located on the body. Methods should be investigated that use RSSI to estimate the distance between “smart” devices that are in close proximity. Presumably these techniques would be analogous to those used in magnetic position and orientation tracking systems [142]. Future research should also consider the possibility that these “smart” garments (embedded with MEMS sensors) may eventually become preferable to utilising the sensors within the smartphone for identifying physical movement. In this scenario, the smartphone may become the “data-hub” or nexus at which all the sensor data is processed. If this should eventuate, it will become necessary to develop new ways for establishing communication between wireless devices that do not require the devices to be manually “paired” every time as this would become a hindrance to their use.

#### 4.4.4. Algorithm Personalisation

“Aggregator” applications such as “HealthKit” [143] (from Apple Inc.) and “S Health” [144] (from Samsung) have emerged that consolidate information from various third-party smartphone applications designed to collect data about different aspects of an individual’s health and wellbeing (e.g., blood pressure; heart-rate; weight; location). These aggregator apps could be leveraged to improve algorithms that estimate physical movement by utilising the individual’s personal information (as additional features that were extracted) to tailor the algorithm’s behaviour so that it accounts for their physiological characteristics (age, current fitness level) as well as their daily-routines, both of which may change over time.

## 5. Conclusions

The smartphone has demonstrated a tremendous amount of capability as a non-invasive monitor of physical movement. The studies referenced herein have shown that when the smartphone’s vast array of sensing components are utilised the device can estimate a variety of physical movements with potentially far reaching applications in healthcare. Further research is required to resolve the issues generated by the multifunctional nature of the device as well as the maturation of smartphone technology to mitigate the limitations imposed by battery capacity. The advent of “smartwatches” which contain MEMS sensors, as well as other items of clothing which may become “smart” (*i.e.*, embedded with electrical components that can transduce movement and communicate with other electronic devices wirelessly) have the potential to dramatically impact future methods for identifying movements. Instead, the smartphone could become the hub to which all data is relayed and processed, rather than solely relying on the sensors within the smartphone to identify physical movement.
